# Foods and Beverages

**Published:** 1888-06

**Authors:** 


### FOODS AND BEVERAGES.

The following extracts from W. O. Atwater's contribution to the May *Century* on the above subject will be found valuable to our readers:

"When we boil bones or scoops of meat or fish, to make a soup we extract considerable of gelatinoids, fats and other substances of them. The gelatine in the soup thus made, like the dried gelatine we buy in packages and use for jellies, is, of course, very valuable. It will not take the place of meat because it cannot do all that is done by the albuminoids which the meat contains. But it does part of their work; and if it cannot make flesh it does what is next best in that it saves flesh forming material from being used up. One moral of this is that bones are worth saving for food. In experimenting to find how much nutritive material is extracted from bones in making soup, as it is ordinarily prepared in the household. Dr. König found that beef bones from which the flesh had been removed, yielded from 6 to 7¼ per cent. of their weight of material, of which about 4⅓ per cent. was fat and the rest nitrogenous matter. That is to say, from a pound of bone about an ounce of nutrative material was obtained, of which three-fourth was fat and the rest gelatinoids and the like. But it must be remembered that the bones which the butcher trims out of meat, or which are left on our tables or in our kitchens, usually have a good deal of adhering flesh. This is apt to amount to several times as much as the material extracted from bone itself."

In speaking of the so-called meat extractives the same writer says: "Just what the extractives do in helping to nourish the body has long been a physiological puzzle. At times they appear to aid digestion. It is certain that they have some effect upon the nervous system. When one is weakened by illness or exhausted by hard work they are wonderfully invigorating. They were formerly supposed to furnish actual nutriment, but the tendency of opinion in later years has been to make them simply stimulants, and the experiments within a short time past have indicated very clearly that they neither form tissue nor yield energy; that, indeed, they practically pass through the body unchanged, and are not food at all in the sense in which we use the word. In other words, when a convalescent invalid drinks his beef tea, or a tired brain worker takes meat extracts with his food, though he is greatly refreshed, thereby, and really benefited, the extractives neither repair his tissue nor furnish him warmth or strength. But in some unexplained way they help him to utilize the other materials of his body and of his food to an extent which, without them he could not do. Beef tea and meat extract are strengthening, not by what they themselves supply, but by helping the body to get and to use strength from other materials which it has."

Pursuing further this subject of foods we take the liberty of extracting what follows from a very excellent article of Shirley Dare in last Sunday's New York *Sun* (May 20):

"Bread and milk have been the food of infancy so long that it is like speaking against the pulpit cushions to object to them. But bread and milk or porridge and milk after a child is four years old should not form the staple food that it does in many strictly kept nurseries. This diet makes plump, fair, stout children, but it also makes stupid ones. It always seems as if girls brought up on this conventional diet had curds for brains. Nervous girls dislike it for they need essence of meats to supply their craving nerve fluids. Milk, too, is so susceptible of taint and so ready for carrying disease that, in ordinary conditions it must be a relief to know how excellently broths take its place. Well-seasoned broth of beef, mutton or fowl, thickened with fine oatmeal or cracked wheat, cerealine or farina, or finely cut vegetables, is an ideal food for children and girls, who are also the better for lean, fresh meats boiled or broiled once a day. * * * *

Boarding schools for both girls and boys do great harm to young constitutions by monotonous, insipid fare. The sedentary life, when two hours' exercise to six or eight hours' study exactly reverses the right proportions for most girls and many boys. is fed on corned beef dinners, with rice or cornmeal as a vegetable, codfish breakfasts, and suppers of hot biscuit, cookies and cheese. The craving and faintness felt by growing girls, especially nervous ones with quick, large brains, is a sign that nature is taxed to its utmost to keep up with study or intense thought, and at the same time perfect physical development. Girls of a sensitive temperament are studying their busiest just at an age when they should be living an easy, active life, learning to grow flowers and embroider them, to sing, to draw, to dance, to converse, how to keep house and having a good time till they are twenty, when they can take up the serious studies in earnest if they choose. The instinct of girls to have a good time in spite of everything is their salvation from the entire nervous wreck which school systems propose for them. A girl can learn and retain more in three hours' study a day than she can in seven, and the mind receives better training by taking up few studies at a time and fastening on them than by the modern public school method of skipping about topics, hardy two days on the same study in a week."

